# Translational role of molecular biomarkers in developing targeted therapies for central nervous system tumors

**DOI:** 10.1016/j.ibneur.2025.11.006

**Published:** 2025-11-07

**Authors:** Jaishriram Rathored, Tanushree Budhbaware, Sushma Chauhan, Mithlesh Kumar

**Affiliations:** a Central Research Laboratory and Molecular Diagnostics, School of Allied Health Sciences, Datta Meghe Institute of Higher Education and Research, Sawangi (Meghe), Wardha, Maharashtra 442001, India; b Amity Institute of Biotechnology, Amity University, Raipur, Chhattisgarh 493221, India

**Keywords:** Central nervous system tumors, Biomarkers, Targeted therapy, Precision medicine, Translational research, Tumor heterogeneity

## Abstract

**Background:**

The identification of biomarkers has led to the discovery of new pathways for precision diagnostics and tailored therapies in neoplasms of the central nervous system, thereby enhancing prognoses due to their diverse anatomical structures.

**Objective:**

This review explores advancements in identifying biomarkers in central nervous system tumors and their application in targeted therapeutic strategies.

**Methods:**

The literature review, utilizing electronic databases like PubMed, Scopus, and Web of Science, emphasized studies on CNS tumors, biomarkers, targeted therapy, and precision medicine, emphasizing clinical trials, validation of biomarkers, and molecular characterization.

**Discussion:**

Advances in liquid biopsy and sequencing have significantly impacted the diagnosis and prognosis of CNS malignancies, leading to targeted therapies and improved biomarker discovery.

**Conclusion:**

Biomarker-driven approaches have significantly improved CNS cancer treatment. Future research should focus on confirming new biomarkers, improving delivery methods, and ensuring wider access to precision drugs to improve patient lifespan and quality of life.

## Highlights

• Precision treatment for CNS malignancies is guided by molecular biomarkers.
• The accuracy of diagnosis and prognosis is improved by biomarker profiling.
• Targeted treatments decrease toxicity while improving results.
• Lab results are translated into clinical care through translational research.
• Minimally invasive tumor monitoring is made possible by liquid biopsies.

## 1 Introduction

The central nervous system (CNS), including the brain and spinal cord, plays an important role in the regulation and regulation of physical function by sending, receiving and integrating neural signals. The development of tumors within the CNS can disrupt these essential processes, which often lead to significant neurological symptoms due to compression, invasion, or shifting of critical neurological structures (Lv and Wang, 2024). CNS tumors can be classified as benign (not neoplastic) or malignant (tumoric). Although benign tumors do not have invasive or metastatic behavior, growth within a limited anatomical room, such as the vault or lumbar canal, can lead to increased intracranial pressure or neural dysfunction due to compression of adjacent neural structures. CNS tumors represent the second most important category of pediatric neoplasms that supplement approximately 4000 new diagnoses in children (Damodharan and Puccetti, 2023).

Brain tumors can be classified into two primary categories: essential and auxiliary. Essential brain tumors start inside the brain and can possibly spread to other parts of the central anxious framework, such as the spinal line. However, they rarely metastasize to distant areas of the body (Louis et al., 2021). In contrast, secondary brain tumors, also known as metastatic tumors, arise when cancer cells from other regions of the body spread to the brain. While primary brain tumors are commonly observed in adults, secondary brain tumors are less frequent in pediatric populations. The distinct nature of primary and secondary tumors is crucial for understanding their pathophysiology and implications for treatment strategies (Liu et al., 2024). Certain inherited genetic syndromes can increase the risk of CNS tumors in offspring; however, in the majority of cases, the etiology remains unknown. CNS tumors are thought to arise from errors that occur during normal neurodevelopment (Hale et al., 2024). During embryonic and early postnatal development, neural cells of the brain and spinal cord undergo rapid proliferation and differentiation. This prepare includes the replication of hereditary fabric, and mistakes in DNA replication or repair components can result in physical transformations. Such changes may disturb administrative pathways controlling cell development and apoptosis, driving to uncontrolled cellular multiplication and tumor arrangement. These hereditary changes ordinarily happen sporadically and are not preventable, underscoring the complex and multifactorial nature of CNS tumorigenesis (Belmonte-Mateos and Pujades, 2021). Because of their intricacy and dismal prognosis, central nervous system (CNS) tumors pose a significant clinical challenge to both juvenile and adult populations. About 20 % of all pediatric malignancies are CNS tumors, making them the second most frequent cancer in children after leukemia (Ostrom et al., 2019). The most common malignant brain tumor in adults is glioblastoma, which accounts for almost 50 % of cases and has a median survival of only 12–15 months even with intensive multimodal treatment. The variability of CNS malignancies, both across and within age groups, exacerbates these difficulties. Recent developments in molecular profiling have revealed important biomarkers that influence diagnosis, prognosis, and treatment response, including MGMT promoter methylation, IDH1/2 mutations, and H3K27M mutations. These discoveries open the door to customized, biomarker-driven treatment plans. We can more effectively develop medicines that are not only focused and successful but also flexible enough to address the particular difficulties of treating CNS malignancies in both children and adults by combining anatomical knowledge with molecular insights across age groups. The danger of neurological impairment is increased and surgical choices are limited by the intricate anatomy of the brain and spinal cord. Additionally, the effectiveness of many systemic treatments is diminished by the blood–brain barrier (BBB), which significantly limits medication delivery (Deeken and Löscher, 2007).

To find pertinent research on biomarkers and targeted treatments for malignancies of the central nervous system (CNS), a thorough literature search was carried out. Literature published between January 2000 and April 2024 was covered by the databases that were searched, which included PubMed, Scopus, and Web of Science. The following Boolean combinations and search phrases were applied: AND ("biomarkers" OR "molecular markers" OR "genetic alterations") AND ("targeted therapy" OR "precision medicine" OR "companion diagnostics") AND ("CNS tumors" OR "brain tumors" OR "glioma" OR "glioblastoma").

1. Original research papers that have undergone peer review, meta-analyses, and systematic reviews
2. Research on molecular biomarkers for CNS malignancies that are important for diagnosis, prognosis, or treatment
3. English-language publications
4. Research on CNS malignancies in adults and children

1. Letters, editorials, or conference abstracts devoid of unique information
2. Research that is not pertinent to CNS-specific biomarkers or focused therapies
3. Publications in languages other than English

**Fig. 1:**
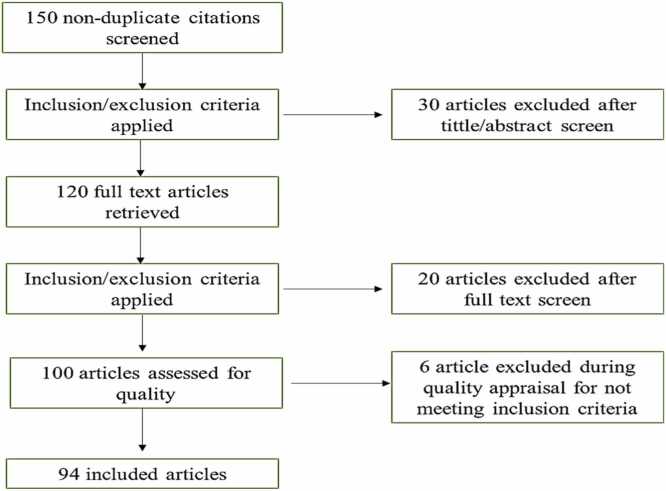
PRISMA tool for reporting review search.

The PRISMA flow diagram outlines given below shows the systematic screening process, depicting the number of records identified, screened, assessed for eligibility, and included in the final synthesis [Fig. 1].

## 2 Molecular landscape of CNS tumors

**Fig. 2:**
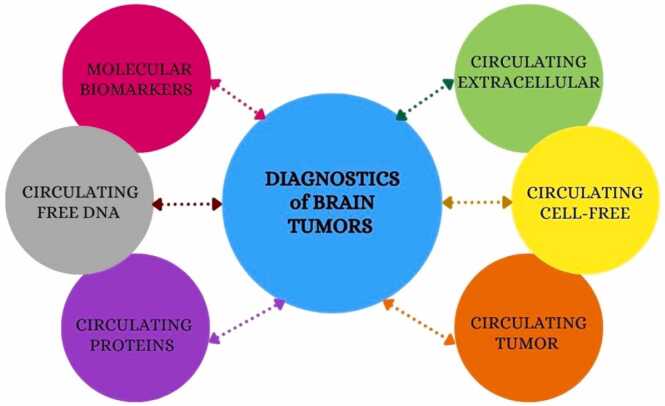
Tumor biomarkers.

Methods for liquid biopsies offer a less intrusive insight into the biology of CNS tumors. Circulating tumor cells (CTCs), cell-free DNA (cfDNA/ctDNA), microRNAs (miRNAs), extracellular vesicles (EVs), and circulating proteins are some examples of these biomarkers. Every class has its own limits but also provides unique insights [Table 1]. We provide a summary of these categories below (Felekkis and Papaneophytou, 2024) [Fig. 2].

**Table 1:** Framework of liquid biopsy biomarkers in CNS tumors.

Biomarker type	Source/origin	Common detection methods	Clinical utility	Key limitations
**Circulating tumor cells (ctcs)**	Tumor cells shed into blood/CSF	EpCAM-based assays, telomerase-based methods, immunomagnetic capture	Prognosis, treatment monitoring	Low abundance, EpCAM-negative glioma cells
**Circulating tumor dna (ctdna)**	Apoptotic/necrotic tumor cells → cfDNA in plasma/CSF	Digital PCR, NGS, ultra-deep sequencing	Mutation profiling, resistance tracking, minimal residual disease	BBB restricts release, low sensitivity in plasma
**Circulating mirnas**	Tumor-derived small RNAs in serum/CSF/EVs	qRT-PCR, sequencing, microarrays	Diagnosis, grading, prognosis, treatment monitoring	Heterogeneity, normalization issues
**Extracellular vesicles (evs)**	Exosomes/microvesicles secreted by tumor cells	Ultracentrifugation, NTA, TEM, flow cytometry	Carry intact RNA/proteins; diagnosis, monitoring	Isolation/standardization challenges
**Circulating proteins**	Tumor-derived or host-response proteins in plasma/CSF	ELISA, mass spectrometry, immunoassays	Prognostic markers (e.g., YKL-40, VEGF), therapy monitoring	Limited specificity, overlap with non-tumor conditions

### 2.1 Biomarkers discovery and validation

#### 2.1.1 Molecular marker

The molecular characterization of central apprehensive framework (CNS) tumors has uniquely changed their classification, determination, and forecast over the past decade. Since the formal consolidation of atomic biomarkers into CNS tumor diagnostics by the World Wellbeing Organization (WHO) in its 2016 classification, a few key biomarkers have developed as crucial to clinical neuropathology (Kristensen et al., 2019). Atomic biomarkers play a basic part within the determination, guess, and restorative stratification of CNS tumors, especially gliomas and glioblastomas. Among the foremost clinically important biomarkers are those included in DNA repair, receptor tyrosine kinase signaling, metabolic pathways, cytoskeletal judgment, telomere support, and tumor silencer capacities (Rajakaruna et al., 2025). MGMT (O6-methylguanine-DNA methyltransferase) promoter methylation is one of the foremost well-established prescient biomarkers in glioblastoma. Located on chromosome 10q26, MGMT encodes a DNA repair enzyme that removes alkyl groups from the O6 position of guanine (Chen et al., 2023). Methylation of the MGMT promoter silences gene expression, impairing the cell's ability to repair temozolomide (TMZ)-induced DNA damage. Clinically, MGMT promoter methylation correlates with increased sensitivity to TMZ and longer overall survival, particularly in secondary glioblastomas where methylation is more prevalent (∼ 75 %) compared to primary glioblastomas (∼ 36 %) (MGMT, 2025). EGFR (epidermal growth factor receptor) is frequently amplified (∼ 40 %) in glioblastomas and is associated with increased tumor aggressiveness. A specific mutant variant, EGFRvIII, characterized by a deletion in the extracellular domain, is constitutively active and ligand-independent. EGFRvIII overexpression is often co-amplified with EGFR and has been variably reported as both a poor and favorable prognostic indicator, depending on therapeutic context. Although EGFR amplification confers initial responsiveness to receptor tyrosine kinase inhibitors, resistance often develops (PDF). A number of systematic reviews and meta-analyses have validated the predictive function of MGMT promoter methylation in glioblastoma. for example, showed a substantial correlation between it and temozolomide responsiveness (Binabaj et al., 2018) and Later meta-analyses have confirmed that in glioblastoma patients after alkylating chemotherapy, MGMT methylation is associated with better overall and progression-free survival (Zhang et al., 2013). Higher-level evidence that MGMT methylation is a reliable predictive biomarker is provided by these pooled analyses.

IDH1 and IDH2 (isocitrate dehydrogenase) mutations are hallmark features of lower-grade gliomas and secondary glioblastomas. These mutations result in the production of the oncometabolite D-2-hydroxyglutarate, which induces epigenetic alterations including global DNA and histone hypermethylation (Zhao et al., 2024). IDH-mutant gliomas typically have better prognoses and increased sensitivity to both radiotherapy and chemotherapy. Immunohistochemistry and sequencing-based methods such as pyrosequencing or ddPCR are routinely used for detection (Weller et al., 2024). GFAP (glial fibrillary acidic protein) is an astrocytic marker frequently used in the histological classification of CNS tumors. Although GFAP expression is commonly elevated in tumor tissue, its detection in serum is limited due to intratumoral heterogeneity and the so-called "sensitivity gap." Nonetheless, serum GFAP levels correlate with tumor burden, necrosis, and IDH status, and GFAP remains a valuable marker for circulating tumor cell identification (Jelski and Mroczko, 2021). TERT (telomerase reverse transcriptase) promoter mutations (C228T and C250T) are frequent in gliomas and lead to reactivation of telomerase, facilitating cellular immortalization. These mutations are not found in normal cells and are considered prognostic biomarkers. Detection through liquid biopsy has shown potential for noninvasively diagnosis and monitoring (Powter et al., 2021). Loss of heterozygosity (LOH), particularly on chromosomes 10q and 22q, is common in gliomas. LOH at 10q frequently involves PTEN, a tumor suppressor that negatively regulates the PI3K/AKT/mTOR pathway (Crespo et al., 2015). Deletion of 22q12.3 leads to loss of TIMP-3, a matrix metalloproteinase inhibitor. LOH on 1p/19q is a hallmark of oligodendrogliomas and predicts favorable outcomes and treatment response. Detection is generally performed using microsatellite PCR-based analysis (Nigro et al., 2001). TP53 mutations are significantly enriched in secondary glioblastomas (up to 90 %) compared to primary glioblastomas (approximately 30 %). Mutant p53 can drive tumorigenesis through deregulation of the mevalonate pathway, enhancing oncogenic enzyme expression. TP53 status can inform therapeutic approaches involving MDM2 inhibitors. Together, these molecular biomarkers are instrumental in advancing the precision medicine approach in CNS oncology, offering improved diagnostic accuracy and guiding personalized therapeutic strategies (Ahmadi et al., 2024) [Table 2].

**Table 2:** Molecular marker in CNS tumor.

Biomarker	Function/Role	Clinical Relevance (with survival data)	Detection Method
**MGMT**	DNA repair	Predictor of TMZ sensitivity; MGMT promoter methylation linked to improved survival (median OS ∼21.7 mo vs. 12.7 mo in unmethylated)	PCR
**EGFR/EGFRvIII**	Receptor tyrosine kinase; constitutively activated	Associated with tumor aggressiveness; variable prognostic value (not consistently correlated with OS); target for TKIs	FISH, IHC, PCR
**IDH1/IDH2**	Metabolic enzymes generate D-2HG	Strong favorable prognosis; IDH-mutant gliomas show prolonged survival (HR for death ∼0.27; median OS 31–36 mo vs. 15 mo in wild-type)	IHC, pyrosequencing, qPCR
**GFAP**	Astrocytic structural protein	Histological marker; serum GFAP levels correlate with tumor burden and necrosis; not strongly linked to OS	IHC, ELISA
**TERT promoter mutations (C228T, C250T)**	Telomere maintenance	Poor prognosis in GBM; TERT mutations often co-occur with IDH status (IDH-wildtype + TERT mutation associated with worse survival)	PCR, Liquid biopsy
**LOH (1p/19q, 10q, 22q)**	Genomic instability; tumor suppressor loss	1p/19q codeletion is hallmark of oligodendroglioma; predicts favorable prognosis and treatment response (median OS ∼ 14 yrs vs. 7 yrs without deletion)	Microsatellite PCR
**TP53**	Tumor suppressor regulates cell cycle	Common in secondary GBM (∼ 90 %); role in prognosis less clear, but may indicate better outcome in low-grade gliomas	IHC, sequencing

### 2.2 Circulating tumor cells

**Fig. 3:**
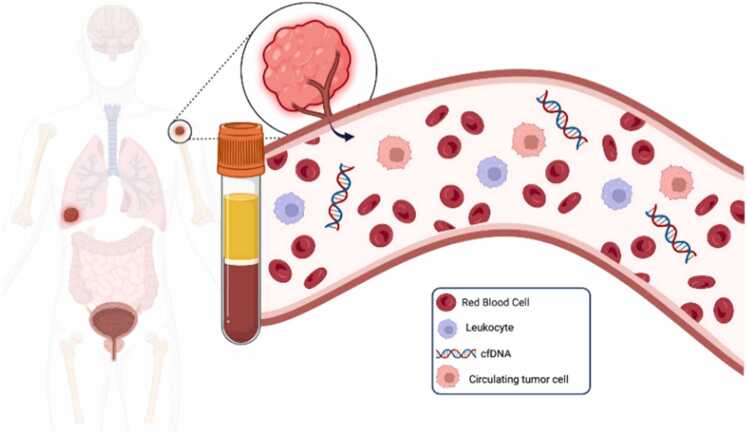
Circulating tumor cells (Eslami-S et al., 2020).

Circulating tumor cells (CTCs) are malignant cells that detach from either primary or metastatic neoplasms and disseminate into various body fluids, including blood, cerebrospinal fluid (CSF), and, in some instances, urine [Fig. 3]. These cells play a critical role in the metastatic cascade by reflecting the invasive potential of epithelial tumors, although their exact relationship to the primary tumor's cellular heterogeneity remains a topic of ongoing investigation. In the context of central nervous system (CNS) tumors, particularly glioblastoma (GBM), the detection and characterization of CTCs have garnered increasing scientific attention (Auwal et al., 2024). Although CNS tumors traditionally exhibit limited extracranial metastasis due to the restrictive nature of the blood-brain barrier, recent studies have confirmed the presence of CTCs in the peripheral circulation of glioblastoma patients. This finding underscores the potential for CTCs to act as surrogates for tumor tissue, giving understanding into tumor science through a negligibly intrusive "fluid biopsy" (Kurdi et al., 2023). CTCs have illustrated prognostic esteem in a assortment of malignancies, such as melanoma, lung cancer, and pheochromocytoma. So also, in glioblastoma, CTCs may educate infection movement and restorative reaction. Eminently, CTC tallies have been watched to diminish altogether taking after chemotherapy, proposing their utility in recognizing tumor movement from treatment-induced changes such as radiation necrosis (Capuozzo et al., 2023).

The molecular profiling of CNS-derived CTCs offers an road for following tumor advancement. Particular genotypic modifications, counting EGFR enhancement and telomerase movement, can be analyzed from CTC tests, possibly uncovering energetic changes related with repeat or treatment resistance (Dejima et al., 2024). Vitally, up to 75 % of glioblastoma patients have been detailed to harbor perceptible CTCs, fortifying their predominance and significance. In any case, a few specialized impediments prevent the schedule clinical application of CTC examination in CNS tumors (Lynch et al., 2020). Conventional CTC location stages regularly depend on epithelial cell grip atom (EpCAM)-based enhancement, which is imperfect for glioblastoma cells that ordinarily need EpCAM expression. Therefore, alternative detection methodologies leveraging telomerase-based assays or tumor-specific genetic markers are under investigation (Eslami-S et al., 2020). while CTCs hold considerable promise as biomarkers for diagnosis, prognosis, and therapeutic monitoring in CNS tumors, their clinical utility is contingent upon the development of more sensitive and specific detection technologies. Further research is essential to establish standardized protocols and validate their role in clinical decision-making (Hsu et al., 2024).

### 2.3 Cell-free DNA

**Fig. 4:**
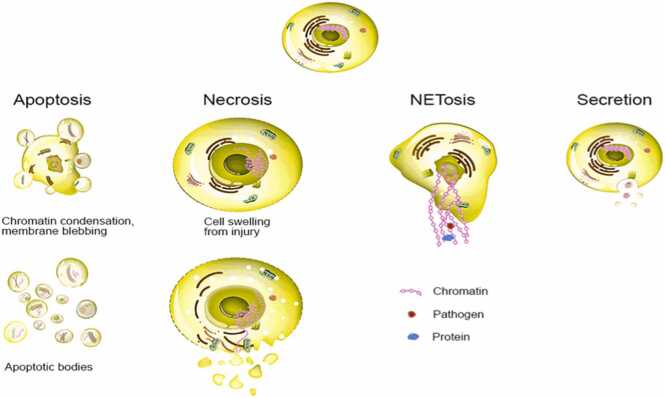
Origin of cfDNA: Secretion, NETosis, necrosis, and apoptosis (cfDNA cell-free DNA & cfDNA Extraction, 2025).

Circulating tumor DNA (ctDNA), a subset of cell-free DNA (cfDNA), consists of short DNA fragments released into the bloodstream through apoptosis or necrosis of malignant cells (Stejskal et al., 2023) [Fig. 4]. While cfDNA in individuals without cancer predominantly originates from apoptotic cells and is generally longer (> 500 bp), ctDNA in cancer patients is characterized by shorter fragments and carries tumor-specific genetic alterations. In the context of primary CNS tumors, the utility of ctDNA as a biomarker is uniquely constrained compared to other solid tumors (Stewart and Tsui, 2018). Despite the elevated levels of ctDNA observed in many extracranial malignancies correlating with tumor burden and progression, ctDNA is typically detected at low concentrations in the serum of patients with gliomas, including glioblastoma. This limitation is partly due to the restrictive nature of the blood brain barrier, which impedes the release of ctDNA into systemic circulation (Yan et al., 2020). Nevertheless, studies have demonstrated the feasibility of ctDNA detection in some CNS tumor patients, including those with astrocytomas and oligoastrocytomas (Kim et al., 2025). The diagnostic yield of ctDNA from plasma is modest; however, cerebrospinal fluid (CSF) has emerged as a more reliable source. Superior sensitivity of ctDNA detection in CSF compared to plasma in patients with primary brain tumors. Furthermore, ctDNA from CSF can more accurately reflect the tumor’s genomic landscape, capturing clinically actionable mutations, mechanisms of resistance, and subclonal heterogeneity (Hickman et al., 2023). The detection of somatic alterations in ctDNA depends critically on both the absolute quantity of ctDNA present and the sensitivity of the analytical platform employed. Technological advancements, including ultra-deep sequencing and digital PCR, have improved detection thresholds, enabling identification of low-frequency variants (Martínez-Vila et al., 2025). Importantly, ctDNA dynamics can serve as a surrogate for tumor progression and therapeutic resistance, offering potential utility in longitudinal disease monitoring and early relapse detection (Elliott et al., 2025).

The presence and detectability of ctDNA in CSF is influenced by the tumor’s anatomical location and histological grade rather than its size. Due to ctDNA's rapid clearance from circulation (half-life ∼ 1.5–2 h), pre-analytical handling is crucial blood or CSF samples should be processed within 3 h and stored at − 80 °C to preserve nucleic acid integrity (Cabezas-Camarero et al., 2025). Although challenges remain, ctDNA represents a promising biomarker in CNS tumors, particularly when sourced from CSF. It offers non-invasive insights into tumor genomics, potentially facilitating personalized treatment strategies and real-time monitoring of disease dynamics (Bartolomucci et al., 2025).

### 2.4 Cell-free miRNA

**Fig. 5:**
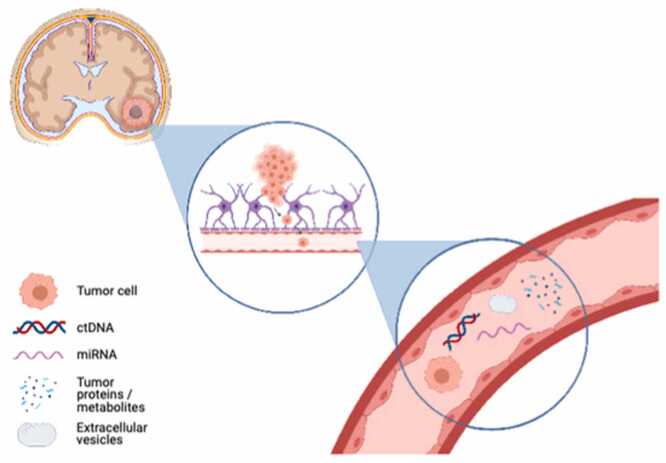
Methods for liquid biopsies are used to diagnose or track tumors in the central nervous system (Bunda et al., 2021).

MicroRNAs (miRNAs) are small (∼22–25 nucleotides), non-coding RNA molecules that post-transcriptionally regulate gene expression by promoting degradation or translational inhibition of target messenger RNAs (mRNAs) [Fig. 5]. Their regulatory functions extend to a wide range of physiological and pathological processes, including cell proliferation, differentiation, apoptosis, and immune responses (Ratti et al., 2020). In the context of central nervous system (CNS) tumors, particularly glioblastoma multiforme (GBM), miRNAs have emerged as key molecular players and promising biomarkers due to their disease-specific expression patterns and stability in biological fluids (Makowska et al., 2023). MiRNAs are differentially expressed in glioblastoma tissues and circulating fluids, and their dysregulation contributes to tumorigenesis, treatment resistance, and clinical outcome. For instance, miR-21, one of the most extensively studied oncomiRs, is consistently overexpressed in both glioblastoma tissues and patient sera (Aloizou et al., 2020). It functions as an anti-apoptotic factor by targeting tumor suppressors and inhibiting caspase-mediated cell death pathways. Experimental silencing of miR-21 has been shown to suppress tumor growth, promote apoptosis, and reduce the proliferative capacity of GBM cells. Notably, miR-21 expression also correlates with glioblastoma grade, overall survival, and treatment response, underscoring its utility as both a diagnostic and prognostic biomarker. Furthermore, miR-21 has been implicated in the regulation of glioblastoma cancer stem-like cells, potentially through Fas ligand signaling pathways (Prasad et al., 2025).

Beyond miR-21, other miRNAs have shown clinical relevance. For example, circulating levels of miR-185 have been reported to be significantly elevated in GBM patients compared to healthy controls, with levels returning to baseline following surgical resection and chemoradiotherapy, suggesting its role as a dynamic biomarker for treatment monitoring (Suvarnapathaki et al., 2024). Conversely, serum levels of miR-125b are frequently downregulated in glioblastoma, providing a potential diagnostic marker. Additional tumor-suppressive miRNAs, including miR-128, miR-485-3p, and miR-342-3p, are also reduced in GBM patient sera and show negative correlations with tumor grade. Their expression levels tend to normalize post-treatment, suggesting utility in tumor grading and therapeutic response assessment (Wu et al., 2013). Importantly, miRNAs are highly stable in biological fluids such as serum, plasma, and cerebrospinal fluid (CSF), and can be accurately quantified using real-time PCR-based techniques. This enables minimally invasive detection of CNS malignancies with high specificity often exceeding 90 % and allows longitudinal monitoring of disease progression or treatment efficacy. Given their distinct expression profiles, diagnostic accuracy, and prognostic potential, miRNAs represent a valuable class of molecular biomarkers for glioblastoma and possibly other CNS tumors (Mestry et al., 2023). miRNA profiling holds significant promise in the clinical management of CNS tumors, especially glioblastoma, by enabling early detection, patient stratification, prognosis determination, and real-time monitoring of therapeutic outcomes. Continued exploration of miRNA signatures and integration into multi-omic diagnostic platforms could further refine their clinical application in neuro-oncology (Seyhan, 2024).

### 2.5 Circulating extracellular vesicles

**Fig. 6:**
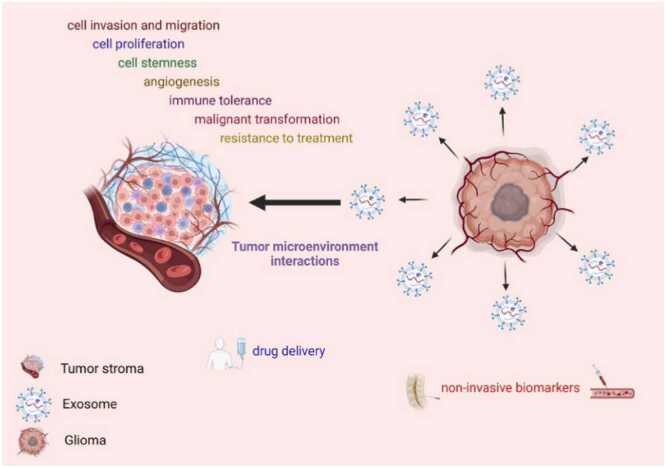
The pathophysiology and therapy of gliomas in relation to exosomes (Skouras et al., 2023).

Extracellular vesicles (EVs), particularly exosomes, are emerging as promising biomarkers for central nervous system (CNS) tumors, including glioblastoma multiforme (GBM), due to their role in tumor progression and intercellular communication (Yang et al., 2024) [Fig. 6]. These nanoscale vesicles (typically 30–100 nm in diameter) are secreted by glioma cells into the tumor microenvironment and peripheral circulation, encapsulating a diverse array of bioactive molecules such as proteins, lipids, mRNAs, microRNAs, and genomic DNA. As such, they offer a non-invasive window into the molecular landscape of CNS tumors (Kuang et al., 2025). EVs derived from glioblastoma cells have been shown to modulate the behavior of surrounding stromal and endothelial cells, enhancing angiogenesis and tumor invasiveness via autocrine and paracrine mechanisms. Notably, they have been implicated in the transfer of oncogenic cargo, including amplified or mutant forms of EGFR, such as EGFRvIII (Choi et al., 2018). In a seminal study by Skog et al., EVs isolated from the serum of glioblastoma patients contained detectable levels of tumor-specific EGFRvIII mRNA, demonstrating their diagnostic potential. Further comparative analyses of circulating EVs in glioblastoma patients versus healthy controls have revealed distinct RNA expression profiles, with elevated concentrations of tumor-derived EVs correlating with tumor burden, progression, and recurrence post-surgical resection. These findings suggest that EVs may serve as dynamic biomarkers for disease monitoring, potentially distinguishing glioblastoma from other CNS pathologies or non-malignant lesions (Yekula et al., 2020).

Technically, EVs are typically isolated from serum using differential ultracentrifugation, density gradient separation, or polymer-based precipitation methods. Characterization is performed through nanoparticle tracking analysis (NTA), transmission electron microscopy (TEM), and immunodetection of canonical surface markers such as ICAM-1 and integrins via flow cytometry or Western blotting (Kowkabany and Bao, 2024). Altogether, CNS tumor-derived EVs represent a compelling avenue for biomarker discovery, offering minimally invasive diagnostic and prognostic tools for glioblastoma and possibly other neuro-oncological conditions (Chen et al., 2021).

### 2.6 Circulating protein

In patients with central nervous system (CNS) tumors, a range of circulating protein (CP) biomarkers have been identified that reflect tumor-specific biological processes and can be used for diagnostic, prognostic, and therapeutic monitoring purposes (Zhou et al., 2024). These protein markers are often detected in plasma, urine, or cerebrospinal fluid (CSF), and their altered expression profiles may correspond to tumor burden, angiogenic activity, and immune responses (Xiao et al., 2020) [Fig. 5]. Early investigations identified several plasma-derived proteins with elevated concentrations in brain tumor patients, including immunosuppressive acidic protein (IAP), α1-acid glycoprotein, α1-antitrypsin, fibronectin, and thrombomodulin-1. These proteins are often associated with systemic inflammation or vascular damage, which are hallmarks of high-grade brain tumors (Kikuchi et al., 1987). Subsequent studies focused on angiogenesis-related biomarkers, with vascular endothelial growth factor (VEGF) emerging as a prominent candidate. VEGF levels are consistently elevated in patients with primary and metastatic brain tumors and have shown correlation with tumor progression and invasiveness (Pathak et al., 2024). Notably, soluble VEGFR-1 (sVEGFR-1), but not sVEGFR-2 or sVEGFR-3, along with basic 70 fibroblast growth factor (bFGF/FGF-2), were found to be elevated in preoperative serum samples from newly diagnosed cases. These angiogenic factors reflect neovascular remodeling within the tumor microenvironment and may offer diagnostic and therapeutic insights (Barleon et al., 2001).

Proteins involved in extracellular matrix (ECM) remodeling, such as matrix metalloproteinases (MMPs) and their inhibitors (TIMPs), also serve as potential diagnostic CP biomarkers. These markers are implicated in tumor invasion and are sometimes correlated with histological grade and tumor stage (Cabral-Pacheco et al., 2020). However, challenges in specificity exist, as several CNS-related proteins such as brain-derived neurotrophic factor (BDNF) and S100B protein can also be detected in healthy individuals. Therefore, while they may contribute to a diagnostic panel, their standalone utility is limited. Inflammatory and immune-related CP biomarkers including interleukin-2 (IL-2), tumor necrosis factor-alpha (TNF-α), transforming growth factor-beta (TGF-β), neural cell adhesion molecule (NCAM), neuropeptide Y (NPY), and chitinase-3-like protein 1 (CHI3L1/YKL-40) have also been studied (Xiong et al., 2024). Among these, YKL-40 has shown strong correlation with both tumor burden and poor clinical outcomes, making it one of the most promising prognostic indicators for glioma patients (Iwamoto et al., 2011).

For prognostic evaluation, CP markers can be broadly divided into tumor-derived factors (e.g., YKL-40, extracellular EGFR, osteopontin) and stress-response proteins (e.g., PAI-1, HGF). Increased serum YKL-40 and extracellular EGFR domain levels have been inversely correlated with overall survival (OS), while elevated PAI-1 and HGF in CSF are associated with reduced progression-free survival (PFS) (Johansen et al., 2006). CP markers are also being explored for therapeutic monitoring, particularly in the context of antiangiogenic treatments such as bevacizumab and irinotecan. Longitudinal analysis has revealed that reductions in circulating VEGF levels post-treatment correlate with improved clinical outcomes, although baseline VEGF concentrations have not consistently predicted response. This underscores the need for dynamic, treatment-responsive biomarkers rather than static baseline measures (Oguntade et al., 2021). Circulating protein biomarkers offer a minimally invasive means to support diagnosis, prognosis, and treatment monitoring in CNS tumors. However, further validation in large, prospective clinical cohorts is essential to refine their clinical utility and integrate them into standardized neuro-oncological practice (Velpula and Buddolla, 2025) [Table 3].

**Table 3:** Clinical utility and limitations of molecular biomarkers in gliomas.

**Biomarker**	**Tumor Type**	**Detection Method**	**Advantages**	**Limitations**	**Sensitivity/Specificity**
**IDH1/IDH2 Mutations**	Glioma (low/high grade)	PCR, NGS, IHC	Strong prognostic marker; associated with better survival	May be absent in high-grade or secondary glioblastomas	Sensitivity: ∼ 85–95 %; Specificity: ∼ 90–95 %
**MGMT Promoter Methylation**	Glioblastoma	Methylation-specific PCR	Predicts response to temozolomide chemotherapy	Heterogeneous expression; may vary across tumor regions	Sensitivity: ∼ 70–85 %; Specificity: ∼ 75–90 %
**1p/19q Co-deletion**	Oligodendroglioma	FISH, NGS	Indicates better prognosis and chemosensitivity	Primarily relevant only in oligodendroglial tumors	Sensitivity: ∼ 85–95 %; Specificity: ∼ 95 %
**H3K27M Mutation**	Diffuse midline glioma	NGS, PCR	Diagnostic for WHO Grade IV tumors; defines distinct entity	Poor prognosis; limited targeted therapies available	Sensitivity: ∼ 90 %; Specificity: > 95 %
**TERT Promoter Mutation**	Glioblastoma, Oligodendroglioma	PCR, NGS	Associated with aggressive tumor behavior and poor prognosis	Prognostic value may vary with co-occurring mutations (e.g., IDH, 1p/19q)	Sensitivity: ∼ 80–90 %; Specificity: ∼ 85–90 %
**EGFR Amplification/Mutation**	Glioblastoma	FISH, NGS, IHC	Targetable alteration; associated with tumor proliferation	Limited success of EGFR-targeted therapies due to intratumoral heterogeneity	Sensitivity: ∼ 65–80 %; Specificity: ∼ 70–85 %
**BRAF V600E Mutation**	Pleomorphic xanthoastrocytoma, ganglioglioma	PCR, NGS	Predictive marker for BRAF inhibitors	Rare in adult gliomas; resistance mechanisms may develop	Sensitivity: ∼ 85–95 %; Specificity: ∼ 95 %

### 2.7 Translational tools and technologies

Advances in translational tools and technologies are revolutionizing cancer diagnostics and personalized therapy by bridging the gap between bench research and clinical application. Liquid biopsy is a promising development that enables real-time monitoring of tumor dynamics through the analysis of circulating tumor DNA (ctDNA), extracellular vesicles (EVs), and cerebrospinal fluid (CSF) markers (Jahangiri, 2024). These biomarkers help detect minimal residual disease, identify actionable mutations, and track treatment resistance in solid tumors. Companion diagnostics and specialized assays co-evolve with targeted therapies, stratifying patients based on molecular profiles and ensuring the right therapeutic is matched with the right individual. Regulatory frameworks now emphasize the co-approval of such diagnostics with new therapeutics, enhancing clinical decision-making and improving patient outcomes (Ye et al., 2025). The integration of bioinformatics and machine learning is transforming the interpretation of complex biological data, accelerating biomarker discovery, and enabling precision oncology. High-throughput omics technologies generate vast datasets that require robust computational tools for meaningful analysis (Zhang et al., 2025). Artificial intelligence (AI) is being leveraged to automate histopathological image analysis, discover novel mutational signatures, and model tumor evolution under therapy. These translational technologies represent a paradigm shift in oncology research and practice, promoting early detection, dynamic monitoring, and individualized therapy (Liu et al., 2025).

### 2.8 Clinical application

By enhancing diagnostic precision and prognostic evaluation by molecular profiling, biomarker-guided diagnosis and classification has revolutionized the therapeutic management of CNS malignancies (Liu et al., 2025). Today, IDH1/2, EGFR, TP53, and H3K27M mutations are often used in diagnostic processes, improving tumor categorization and directing therapeutic approaches. In order to enhance effectiveness and reduce toxicity, doctors are increasingly using predictive biomarkers for therapeutic response to customize patient-specific therapies (Optimized, 2025). Targeted treatments for IDH-mutant gliomas are among the most promising translational applications. In patients with recurrent or progressing gliomas, the small-molecule inhibitor ivosidenib (AG-120), which specifically targets mutant IDH1, has shown therapeutic efficacy. The therapeutic promise of biomarker-driven therapies in neuro-oncology was highlighted by a phase I clinical trial that found ivosidenib was well tolerated and resulted in sustained disease stabilization in a subset of patients with IDH1-mutant gliomas. In a similar vein, early-phase studies of vorasidenib, a dual IDH1/2 inhibitor, have demonstrated promising outcomes, including long-lasting disease control and positive safety profiles (Hegi et al., 2005). These studies demonstrate how the natural history of malignancies that are thought to be incurable can be changed by molecularly directed treatment. Clinical data backs the application of biomarker-guided approaches for various CNS malignancies in addition to IDH inhibitors. For instance, BRAF inhibitors like vemurafenib or dabrafenib can be used to target BRAF V600E mutations in pleomorphic xanthoastrocytomas and gangliogliomas, frequently leading to significant tumor regression. Similarly, MGMT promoter methylation continues to be a potent predictive biomarker for glioblastoma response to temozolomide, impacting the choice of frontline treatment (Bronkhorst and Holdenrieder). Further demonstrating the difficulty of intratumoral heterogeneity in CNS malignancies are trials examining immunotherapies and EGFR-targeted treatments (such as depatuxizumab mafodotin), which have produced inconsistent but encouraging results. VEGF-guided anti-angiogenic therapy with bevacizumab has also been incorporated into recurrent glioblastoma treatment plans, where it improves symptoms and increases progression-free survival (Platten et al., 2021). All things considered, these illustrations demonstrate how biomarker-guided treatments are used in CNS oncology. By incorporating these focused strategies into standard oncology procedures, precision medicine paradigms that customize treatments to each patient's unique tumor biology and characteristics are made possible, eventually leading to better survival and quality of life.

### 2.9 Challenges and future perspectives

Despite advancements in cancer biology and targeted therapies, challenges persist in precision medicine in oncology. Resistance mechanisms, both intrinsic and acquired, undermine the efficacy of current therapies. Tumor cells often evolve under therapeutic pressure, activating alternative pathways, genetic mutations, or compensatory mechanisms (Liu et al., 2024). The tumor microenvironment (TME) plays a crucial role in tumor progression and resistance. An emerging strategy combines cell-autonomous targets with microenvironmental interventions for a more holistic and durable therapeutic effect (Fang et al., 2025). Clinical practice and research must adopt integrated approaches, including multi-omic profiling, real-time tumor evolution monitoring, and adaptive trial designs. Predictive biomarkers are needed to stratify patients and anticipate resistance development. Collaborative efforts among clinicians, researchers, and data scientists are essential for translating discoveries into bedside applications. Precision oncology frameworks are dynamic, patient-specific, and capable of overcoming resistance through personalized treatment strategies (Sonkin et al., 2024). Finding biomarkers and integrating them into established clinical procedures are two of the biggest obstacles. Despite being proven biomarkers, IDH1/2 mutations and MGMT methylation are less comparable across institutions due to variations in testing methods (PCR, pyrosequencing, methylation-specific PCR, NGS), interpretation, and reporting. Furthermore, there are differences in access due to the wide variations in national payment practices for molecular diagnostics. It is impossible to properly utilize these indicators to maximize therapy selection in the absence of standardized protocols for test standardization and budgetary coverage. Parallel biomarker-drug approval processes, worldwide consensus initiatives, and integration into health-economic analyses are all necessary to close these gaps.

Disparities in access to biomarkers provide a serious obstacle to the equitable use of precision medicine, in addition to biological and technical challenges. Accessibility for patients in low- and middle-income nations is restricted by the high prices of sophisticated molecular testing (such as NGS and methylation profiling) and the concentration of specialist labs in high-income areas. Patients' access to biomarker-driven diagnostics and tailored medicines is frequently influenced by institutional capacity and insurance coverage, even in healthcare systems with abundant resources. Even when there are more effective precision techniques available, patients lacking access may be forced to use traditional therapies, which can worsen result disparities. Global initiatives to lower assay prices, encourage knowledge transfer, and develop infrastructure for biomarker testing in disadvantaged areas are necessary to address these issues.

## 3 Conclusion

Fluid biopsy integration in neuro-oncology is progressing quickly, with prove showing its potential to revolutionize the field through progressions in discovery advances and experiences into CNS tumor science. This innovation upgrades early determination, real-time treatment direction, and understanding results. Regional and socioeconomic disparities in biomarker access must also be addressed in future initiatives. For precision oncology to be successful in the real world, it is imperative that precision diagnoses and treatments be not restricted to specific groups. This disparity might be closed and biomarker-driven treatment made more accessible through tactics including multinational partnerships, decentralized molecular diagnostic hubs, and subsidized testing initiatives. Future ponders ought to center on approving novel biomarkers, moving forward focused on conveyance frameworks, and guaranteeing broader get to accuracy solutions to move forward understanding life span and quality of life. In order to promote substantial patient benefit globally, future frameworks must provide equal weight to the identification of new biomarkers as well as their standardized validation, incorporation into WHO and trial-based processes, and reimbursement system.

## 4 Clinical and research recommendations

Transforming biomarker discoveries into successful treatments for CNS malignancies requires a methodical, multidisciplinary approach. Clinically, validated indicators like MGMT methylation, 1p/19q co-deletion, and IDH1/2 mutations should be used to guide diagnosis, prognostication, and treatment selection. Liquid biopsy methods can allow for individualized, real-time treatment adaption. To guide customized treatment, molecular profiling should be routinely implemented at diagnosis and recurrence. Larger multicenter studies are needed for future research to verify new biomarkers and evaluate their prognostic usefulness in different patient populations. AI techniques like generative adversarial networks (GANs) should be extended to improve biomarker discovery workflows and predict gene expression dynamics. Preclinical and clinical research should focus on integrating cell-autonomous and micro environmental targets for more efficient treatment plans. Bridging the gap between molecular understanding and clinical application is a significant problem and a promising opportunity. Parallel biomarker-drug approval processes, worldwide consensus initiatives, and integration into health-economic analyses are necessary to close these gaps and maximize therapy selection.

## 5 Discussion

Central anxious framework (CNS) tumors speak to a imposing clinical and helpful deterrent owing to their perplexing organic characteristics, anatomical restrictions, and dreary forecasts, particularly in forceful cancers such as glioblastoma multiforme (GBM) (Louis et al., 2021). Later breakthroughs in molecular diagnostics have altogether changed the classification and administration of these tumors, transitioning from conventional histological systems to those educated by atomic bits of knowledge (Baugh et al., 2021). Basic hereditary, epigenetic, and circulating biomarkers have been recognized and completely characterized, in this manner progressing demonstrative exactness, advising helpful procedures, and empowering real-time checking of illness movement (Rahat et al., 2020). A striking progression has been the joining of atomic markers such as IDH1/2 transformations, MGMT promoter methylation, EGFR amplification/mutations, and 1p/19q codeletion into the symptomatic criteria for gliomas, as laid out by the WHO CNS tumor classifications. These biomarkers bestow significant prognostic and prescient importance, exemplified by the strong prescient capacity of MGMT methylation status with respect to temozolomide adequacy (Stupp et al., 2005), whereas IDH transformations categorize a unmistakable subset of gliomas related with more favorable clinical results and one of a kind epigenomic characteristics (Wanis et al., 2024). Crucially, current WHO classifications of CNS malignancies, where molecular profiling is necessary for an appropriate diagnosis, have already been influenced by the inclusion of biomarkers such as MGMT methylation and IDH mutations. To illustrate its direct influence on ordinary neuropathology, the 2021 WHO CNS classification, for instance, requires IDH status in order to distinguish glioblastoma from astrocytoma. Similarly, in glioblastoma procedures, adjuvant temozolomide usage is guided by MGMT methylation. Translation into actual clinical workflows is still patchy, though. Patients are increasingly being stratified in clinical trial designs according to their biomarker status; nevertheless, uniform implementation is hampered by assay variability among laboratories, a lack of established cut-off levels, and variable payment policies. These obstacles restrict fair access, especially in low- and middle-income environments with inadequate infrastructure for molecular testing. Nevertheless, their routine applicability is hindered by the invasive nature of surgical biopsies and resection processes. The advent of liquid biopsy technologies, encompassing circulating tumor cells (CTCs), circulating tumor DNA (ctDNA), microRNAs (miRNAs), extracellular vesicles (EVs), and circulating proteins (CPs), constitutes a crucial advancement in neuro-oncology (Netti et al., 2025) [Fig. 4]. To guarantee repeatability and specificity, the biomarker must be analytically validated in a larger, well-characterized cohort. Creation of a standardized assay with specified sensitivity and specificity parameters (such as PCR-based, IHC, or NGS). Clinical validation is the process of showing a correlation with therapy results through prospective research or retrospective examination of data from clinical trials. CLIA compliance and submission to regulatory agencies such as the FDA for companion diagnostic designation are both part of the regulatory approval process. Incorporation into healthcare workflows with clear usage standards and algorithms for clinician decision-making. These biomarkers afford a minimally invasive approach to obtaining tumor-derived molecular information and hold considerable potential for early detection, patient stratification, treatment monitoring, and identification of minimal residual disease or recurrence. Establish the assay's sensitivity, repeatability, and dependability in identifying the biomarker in clinical samples through analytical validation. Clinical Validation: Use prospective and retrospective cohort studies to establish a relationship between biomarker expression and clinical outcomes. Regulatory clearance: Ensure compliance with companion diagnostic requirements by submitting validation data to regulatory organizations (such as the FDA) for clearance. Integration into Clinical Trials: To show clinical value, use the biomarker to evaluate response in therapy trials or to stratify patients. Implementation: Create economical, standardized testing procedures that clinical labs can employ on a regular basis. There are still a number of practical obstacles in spite of encouraging results. To guarantee data consistency, sample collection and processing must be standardized among centers. Large-scale, multi-center clinical studies are also necessary for validation, but they present logistical and legal difficulties. Prior to these biomarkers being incorporated into standard clinical practice, it is also imperative to reduce inter-laboratory variability, ensure cost-effectiveness, and harmonize assay procedures. CTCs, previously regarded as having limited relevance in CNS tumors due to the impermeability of the blood-brain barrier (BBB), have now been observed in the peripheral circulation of glioma patients, revealing the intricate relationship between tumor biology and vascular integrity (Bronkhorst and Holdenrieder). However, the heterogeneity of CTC phenotypes, alongside the technical challenges associated with their enrichment and detection, necessitates enhancements to existing platforms to improve sensitivity and specificity. Extracellular vesicles (EVs) present another promising class of biomarkers, particularly due to their ability to carry intact nucleic acids and proteins that reflect the tumor's molecular profile. Nevertheless, the technical intricacies involved in EV isolation, purification, and characterization continue to impede their broader clinical implementation (Ghosh et al., 2024). Liquid biopsy techniques for CNS malignancies have a number of drawbacks that prevent widespread clinical application, despite their potential. For example, compared to extracranial malignancies, the blood–brain barrier limits sensitivity and frequently results in a low ctDNA production in plasma. Extracellular vesicle (EV) separation is also a technically challenging process; existing ultracentrifugation and precipitation techniques are not repeatable and frequently co-isolate non-tumor vesicles, which makes subsequent analysis more difficult. Cerebrospinal fluid (CSF)-based assays are becoming more popular as a means of overcoming these obstacles since CSF more accurately represents the tumor microenvironment and has a better sensitivity for identifying genetic changes. Additionally, the identification of low-frequency variations from restricted ctDNA inputs is becoming better because to developments in ultra-sensitive sequencing platforms like digital PCR, error-corrected next-generation sequencing, and single-molecule sequencing. To improve purity and yield, microfluidic and immunoaffinity-based EV separation techniques are also being refined, which might lead to the development of standardized procedures for clinical use. By using these tactics, present restrictions may be lessened and the transition of liquid biopsy from experimental settings to standard neuro-oncology therapy could be accelerated. Precision oncology in CNS cancers is made possible by the molecular pathways covered, including EGFR amplification, IDH1/2 mutations, and MGMT promoter methylation. Crucially, MGMT promoter methylation is one of the most accurate predictive indicators in the treatment of glioblastoma, as supported by systematic reviews and meta-analyses. For instance, MGMT-methylated tumors consistently react better to temozolomide, exhibiting considerably superior survival outcomes when compared to unmethylated instances, according to pooled analyses by Binabaj et al. and Zhao et al. The case for routine MGMT testing in clinical practice is strengthened by the inclusion of such findings (Binabaj et al., 2018; Zhang et al., 2013). For example, MGMT methylation status influences temozolomide response (Hegi et al., 2005), and IDH1 inhibitors such as ivosidenib are being investigated in gliomas (Mellinghoff et al., 2021). Despite their limited success in treating glioblastoma, EGFR-targeted treatments are still developing thanks to new compounds and delivery methods (Taylor et al., 2012). Together, these revelations provide credence to the logical development of biomarker-guided therapeutic approaches. Real-time, non-invasive monitoring of pathway activation and tumor progression in CNS malignancies is made possible by the incorporation of liquid biopsy panels, such as circulating tumor DNA (ctDNA), extracellular vesicles, and tumor-derived RNA (Liu and Weng, 2022). For instance, MGMT methylation or IDH1 mutations found in plasma or CSF can be used to monitor the emergence of resistance and inform treatment choices (Panditharatna et al., 2018, Miller et al., Sep. 2019). By enabling early detection, minimal residual disease evaluation, and dynamic response tracking, these techniques convert molecular insights into clinically useful plans for individualized and flexible patient care. Generative adversarial networks (GANs) offer a novel computational approach for gene expression research that can enhance model training and biomarker detection by generating realistic synthetic data. For instance, in situations when real-world samples are scarce, GANs can be employed to create synthetic datasets for uncommon biomarkers in CNS malignancies. This method enhances biomarker discovery workflows, improves classifier robustness, and reduces data imbalance. Furthermore, by simulating tumor heterogeneity, GAN-based models enable researchers to predict resistance mechanisms and pinpoint treatment weaknesses. Recent research demonstrates how GANs may help with feature extraction and treatment response prediction by replicating high-dimensional single-cell or bulk RNA-seq profiles. Despite these benefits, there are significant ethical issues with using GANs in biomarker research. To prevent misleading or physiologically implausible outcomes that might jeopardize therapeutic development, synthetic data production needs to be strictly monitored. Informed permission, data ownership, and openness are crucial issues for preserving public confidence and scientific integrity in AI-driven research. For GANs to be safely translated into clinical settings, it will be essential to set explicit rules for their responsible usage that strike a balance between innovation and responsibility (Marouf et al., 2020). According to recent multidisciplinary research, GANs can replicate high-dimensional gene expression profiles, which helps with feature extraction and treatment response prediction (Zhang et al., 2021). Thus, using GAN-based models could hasten the creation of biomarker-guided treatments for CNS malignancies. Our results demonstrate the potential of combining biomarkers that represent microenvironmental elements like immune infiltration and angiogenesis as well as tumor-intrinsic (cell-autonomous) changes like EGFR amplification and IDH mutations (Heterogeneity, 2025). For instance, IDH1 mutations propose dual-targeted approaches since they influence the immune milieu in addition to driving carcinogenesis (Platten et al., 2021). Likewise, the use of anti-angiogenic drugs such as bevacizumab in glioblastoma is supported by VEGF expression, a marker of angiogenesis (Chinot et al., 2014). Synergistic treatments that target tumor cells and their supporting habitats can be made possible by developing treatment plans that incorporate these biological findings.

While liquid biopsy integration in neuro-oncology remains in its early phases, growing evidence underscores its potential to revolutionize the field through advancements in detection technologies and insights into CNS tumor biology, thereby enabling precision medicine that enhances early diagnosis, real-time treatment guidance, and patient outcomes (Bao et al., 2024).

## CRediT authorship contribution statement

**Jaishriram Rathored:** Writing – review & editing, Writing – original draft, Visualization, Validation, Supervision, Resources, Project administration, Methodology, Investigation, Formal analysis, Data curation, Conceptualization. **Sushma Chauhan:** Writing – review & editing, Visualization, Methodology, Formal analysis, Data curation. **Tanushree Budhbaware:** Writing – original draft, Visualization, Software, Resources, Methodology, Formal analysis, Data curation. **Kumar Mithilesh:** Writing – original draft, Visualization, Software, Methodology, Formal analysis, Data curation.

## Ethics approval and consent to participate

Not Applicable.

## Consent for publication

Each author approved and gave their consent for the publication.

## Funding

There is no funding used for this review.

## CTRI Registration

Not Applicable.

## Conflicts of Interest

The authors declare no conflicts of interest.

## Data Availability

Not applicable
